# A geographic cline induced by negative frequency-dependent selection

**DOI:** 10.1186/1471-2148-11-256

**Published:** 2011-09-14

**Authors:** Yuma Takahashi, Satoru Morita, Jin Yoshimura, Mamoru Watanabe

**Affiliations:** 1 Division of Ecology and Evolutionary Biology, Graduate School of Life Sciences, Tohoku University, 6-3, Aoba, Aramaki, Aoba, Sendai, Miyagi 980-8578, Japan; 2 Department of Systems Engineering, Shizuoka University, Naka, Hamamatsu 432-8561, Japan; 3 Department of Environmental and Forest Biology, State University of New York College of Environmental Science and Forestry, Syracuse 13210, New York, USA; 4 Marine Biosystems Research Center, Chiba University, Kamogawa, Chiba 299-5502, Japan

## Abstract

**Background:**

Establishment of geographic morph frequency clines is difficult to explain in organisms with limited gene flow. Balancing selection, such as negative frequency-dependent selection (NFDS), is instead suggested to establish a morph frequency cline on a geographic scale at least theoretically. Here we tested whether a large-scale smooth cline in morph frequency is established by NFDS in the female-dimorphic damselfly, *Ischnura senegalensis*, where andromorphs and gynomorphs are maintained by NFDS.

**Results:**

We found a large-scale latitudinal cline in the morph frequency: andromorph frequency ranged from 0.05 (South) to 0.79 (North). Based on the empirical data on the numbers of eggs, the number of ovariole, abdomen length and latitude, the potential fitness of andromorphs was estimated to be lower than that of gynomorphs in the south, and higher in the north, suggesting the gene-by-environment interaction. From the morph-specific latitudinal cline in potential fitness, the frequency of andromorphs was expected to shift from 0 to 1 without NFDS, because a morph with higher potential fitness wins completely and the two morphs will switch at some point. In contrast, NFDS led to the coexistence of two morphs with different potential fitness in a certain geographic range along latitude due to rare morph advantage, and resulted in a smooth geographic cline of morph frequency.

**Conclusion:**

Our results provide suggestive evidence that the combination of NFDS and gene-by-environment interaction, i.e., multi-selection pressure on color morphs, can explain the geographic cline in morph frequency in the current system.

## Background

Patterns of geographic variation in phenotypes and genotypes may provide evidence for selection [1-4]. Geographic clines in quantitative traits caused by environmental gradients have been reported for many species [2,5]. Those are typically smooth due to gradual changes in selection in relation to environmental factors such as temperature [2]. Clines are also observed in morph (or allele) frequencies in species with genetic polymorphisms in both natural and laboratory systems [3,6]. In these cases, the fitness advantage of each morph differentially changes with environmental gradient (i.e., gene-by-environment interaction), and reverses across an equilibrium (balancing) point, where each phenotype has equal fitness [6]. Then, theoretically, morph frequency is expected to show a steep cline (stepwise pattern) across an equilibrium point in the absence of other evolutionary forces antagonistic to local selection [1,6].

A smooth cline in morph (or allele) frequency has been suggested to be a product of two conflicting forces: (i) "local selection," which drives every population to be uniquely adapted to its local environment, and (ii) "gene flow," which tends to make all populations uniform [6]. Given that the gene flow occurs among adjacent populations, the clines in morph frequency established by gene flow are expected to be steep and to be seen only over a relatively short distance [1] (cf. [3]), indicating that small-scale clines in morph frequency (e.g., altitudinal cline and cline in a habitat [7,8]) can be explained by gene flow (dispersal by individuals) among patches with different environments. In practice, morph frequency clines established by gene flow have often been observed at small scales [e.g., [9-11]]. Therefore, in organisms with low dispersal ability, the establishment of geographic-scale morph frequency clines (e.g., latitudinal cline [12]) with gene-by-environment interaction is hardly explained due to a shortage of gene flow [6]. The establishment of a smooth geographic cline in morph frequency in a species with limited or no gene flow (dispersal) is a paradox that needs some other mechanisms other than the balance with gene flow and selection.

Smooth geographic-scale clines in morph frequency over a few hundred kilometers have been reported for many animals and plants, such as floral polymorphisms in narcissus [13,14], wing pattern polymorphisms in ladybird beetles [12], color pattern polymorphism in grasshopper [15], and color polymorphisms in damselflies [16-20]. Some of these clines appear to be difficult to explain by the effects of gene flow, since gene flow is highly effective at relatively small scales in these species [21,22] (but see [23-26]). Instead, "balancing selection," which maintains genetic variation within a population, may be a plausible explanation for the geographic-scale clines to be established and maintained [6]. For example, negative frequency-dependent selection (NFDS), in which rare morphs are favored by selection, and overdominant selection, in which heterozygotes have higher fitness than either homozygote, is theoretically predicted to establish clinal variation in allele (morph) frequency [6,27].

In the case of NFDS, a rare morph becomes advantageous against a common morph. Therefore, a low potential fitness of a rare morph can be compensated by 'rare' morph advantage because of NFDS. This results in the range of coexistence with varying morph frequency along the geographic gradient. In contrast, without NFDS, such compensation does not work and a morph with a higher potential fitness becomes fixed at any location, resulting in a step-cline from one morph to another along a geographic gradient. However, the underlying mechanisms of geographic clines in morph (or allele) frequency are little understood in real examples in the wild, probably because balancing selection among many fitness components has not been sufficiently tested in each polymorphic system.

In some polymorphic species with gene-by-environment interaction, the ranges of morph frequency are narrow due to limitation of their distribution range, and consequently morph frequency cline does not extend beyond an equilibrium point (1:1). For example, in Australian grashopper *Phaulacridium vitatum*, the frequency of striped morph smoothly changes with latitude at geographic scale, but the increase of striped morph frequency was restricted from 10 to 45% within species distribution ranges [15]. Because striped morphs that may be more adaptive than another morphs at opposite side of equilibrium is never supplied by gene flow due to limitation of their distribution, gene flow cannot explain the establishment of the cline. This also suggests that gene flow is not enough to explain the geographic cline in morph frequency in nature.

Genetically determined female polymorphisms occur widely in damselflies and dragonflies [28,29]. One morph exhibits the same color as the conspecific male (andromorph), while the other morph shows a different color from the males (gynomorphs). Previous studies reported that morph frequencies vary geographically [16-20,30]. However, mechanisms behind this phenomenon have not yet been clarified (cf. [19]). In polymorphic damselfly systems, NFDS derived from frequency-dependent male harassment, which hinders females from oviposition and foraging, underlies the coexistence of multiple female morphs [31-34]. For the female-dimorphic damselfly *Ischnura senegalensis*, we tested and confirmed the symmetric NFDS on female color morphs [34]. In this system, male preference is reversed with the morph frequencies and exactly symmetric at the equal frequency (= 0.5) of each morph. We here expect theoretically the 1:1 equilibrium morph frequency if the two morphs have equivalent potential for reproductive performance [34]. Thus, in damselfly system, NFDS is one of a possible mechanism maintaining morph frequency cline in a geographic scale.

In coenagrionid damselflies, the effect of gene flow on the establishment of geographic cline in morph frequency may not be important in some cases. In some species, adult dispersal is typically limited [21,22] and most populations are isolated from one another. In such cases, gene flow (by dispersal of adult individuals) would be too small to produce a geographic cline, even though there are some exceptions [23-26]. According to Endler [6], morph frequency cline along environmental gradients is likely to be independent of the effect of gene flow when balancing selection is acting. As explained later, the gene flow between populations is highly limited in the current system. Thus we can rule out the gene flow over the geographic scale in our analyses. Here we investigated the morph frequency cline at a geographic scale and the morph-specific latitudinal cline in potential fitness (gene-by-environment interaction) in *I. senegalensis*. Then we tested whether the combination of the differential clines in potential fitness and the symmetric NFDS [33,34] can explain a smooth geographic cline in morph frequency along the latitudinal gradient, using the field data of potential fitness for each morph. In the current damselfly system, morph-frequency clines can be solely explained by the balance between the differences in potential fitness and the rare morph advantages caused by NFDS without assuming any gene flow.

## Results

### Latitudinal cline in morph frequency

**Figure 1:**
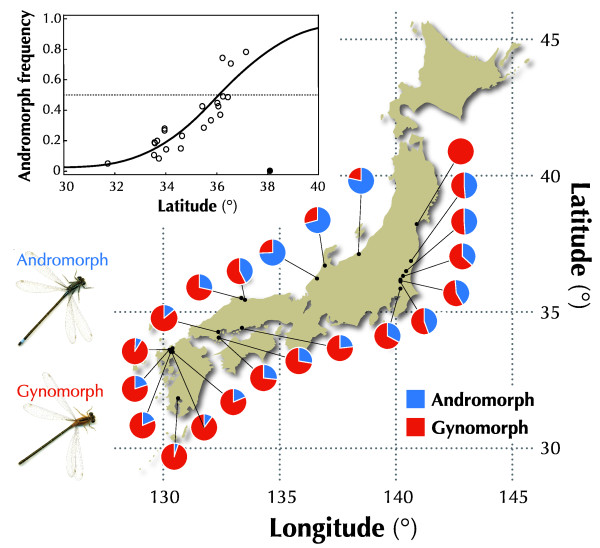
**Latitudinal cline in morph frequency**. Morph frequencies for each local population were shown as a pie chart (blue: andromorph; red: gynomorph). The frequency of andromorphs increased with latitude. The inset Figure shows the logistic regression with the latitude (*t *= 8.15, *df *= 21, *P *< 0.001), excluding the northernmost population (solid plot).

A smooth latitudinal cline was observed in the female morph frequency over a distance of 1100 km in Japan (Figure 1 and Additional file 1 Table S1). The frequency of andromorphs in a local population ranged from 0.05 (South) to 0.79 (North). One exception was the northernmost population where no andromorphs were found (0.00 ratio). We suspect that individuals which carrying the andromorphic (recessive) allele have not yet been introduced given that the local population has established in a newly created (1-year-old) pond (i.e., founder effect). The logistic regression analysis excluding the northernmost population showed that the morph frequency became 0.5 at the northern latitude of 36 degrees.

### Morph-specific latitudinal cline in potential fitness

We here estimated the potential fitness of andromorphs and gynomorphs along latitude, based on the empirical data on the length of abdomen, the number of ovariole, egg size and latitude, assuming the gene-by-environment interaction. From this theoretical analysis, we derived the expected latitudinal cline in morph frequency, as follows.

**Figure 2:**
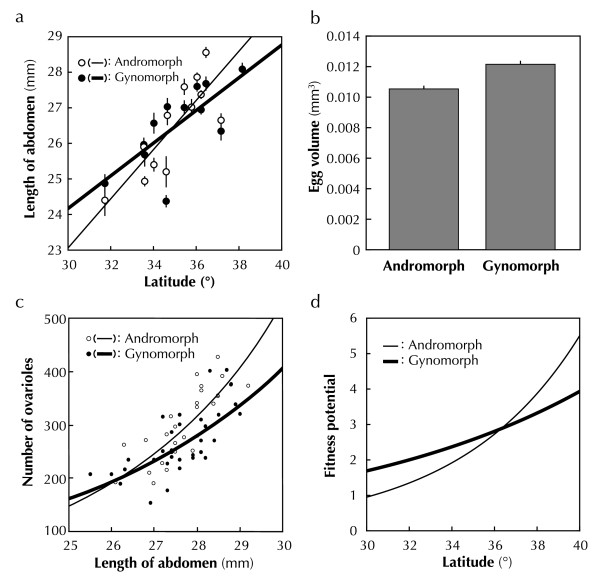
**Latitudinal changes in fitness-related traits**. (*a*) The mean length of abdomen increased with latitude (*t *= 4.618, *df *= 24, *P *< 0.001), although no significant effect of morph or interaction between morph and latitude are found (*t *= 1.244, *df *= 24, *P *= 0.227, *t *= -1.255, *df *= 24, *P *= 0.223). The error bars indicate ± S.E. (*b*) The average volume of mature eggs in andromorphs was significantly smaller than that in gynomorphs (*t *= 6.158, *df *= 22, *P *< 0.001). Error bars indicate ± S.E. (*c*) The number of ovarioles varied significantly with the abdomen length (GLM: *Z *= 16.119, *df *= 59, *P *< 0.001) and morph (GLM: *Z *= 2.621, *df *= 59, *P *= 0.009). The interaction between morph and abdominal length was also significant (GLM: *Z *= -2.808, *df *= 59, *P *= 0.005). (*d*) The potential fitness of gynomorphs was higher than that of andromorphs in the south, and the reverse was true in the north, crossing at approximately 36° latitude (equation (3)). The estimated equations are: *f*(*A*,*l*) = 0.0027 exp(0.584 + 0.176*l*) and *f*(*G*,*l*) = 0.02 exp(0.190 + 0.084*l*).

The mean length *x_i _*(mm) of the abdomen increased linearly with latitude *l *for each morph *i *(Figure 2a), such that

(1) $$x_{i}=b_{i}+a_{i}l\;\left(i=\text{andromorph}\text{(A)}\;\text{or}\;\text{gynomorph}\;\text{(G)}\right),$$

where *a_i _*and *b_i _*are constant parameters. The estimated equations for andromorphs (*x*_A_) and gynomorphs (*x*_G_) were *x*_A _= 2.298 + 0.692*l *(*F *= 21.4, *df *= 10, *r*^2 ^= 0.682, adjusted *r*^2 ^= 0.650, *P *< 0.001) and *x*_G _= 10.366 + 0.460*l *(*F *= 11.98, *df *= 11, *r*^2 ^= 0.521, adjusted *r*^2 ^= 0.468, *P *= 0.005), respectively.

The egg volume was constant irrespective of latitude (*t *= -0.277, *df *= 22, *P *= 0.785), but the effect of morph on egg size was significant (*t *= 6.024, *df *= 22, *P *< 0.001, Additional file 2 Figure S1). The mature eggs in andromorphs (*s*_A _= 0.0106 ± 0.0001 mm^3^) were significantly smaller than those in gynomorphs (*s*_G _= 0.0122 ± 0.0002 mm^3^) (two-sample *t*-test, *t *= 6.158, *df *= 22, *P *< 0.001, Figure 2b).

For each morph, females with longer abdomens exhibited statistically larger number of ovarioles in the ovaries (Figure 2c, see also Additional file 3 Figure S2). In the damselflies, the potential ability for egg production, which correlates with the number of ovarioles [35], is the most important component of potential fitness [28,36,37]. Therefore, assuming that the relationship between the length of the abdomen and the number of ovarioles does not vary with latitude, the average number *y_i _*of ovarioles in ovaries for each morph was given as

(2) yi=ci exp(dixi),

where *c_i _*and *d_i _*are positive constants. We used exponential curve fitting because the space in the abdomen that contains the ovarioles increases exponentially rather than linearly with the length of abdomen. The Akaike Information Criterion (AIC) also suggested that an exponential regression fit better than a linear regression for both andromorphs (linear: AIC = 193.37; exponential: AIC = -100.94) and gynomorphs (linear: AIC = 257.96; exponential: AIC = -122.91). The estimated curves were *y*_A _= 0.259 exp(0.254*x*_A_), and *y*_G _= 1.640 exp(0.184*x*_G_), respectively.

The number of ovarioles directly determines potential fecundity (egg development ability) [28,36,37], and the survival probability of eggs and larvae depends on egg size *s_i _*[38-40]. In the current study, to simplify the model, we assumed a proportional relationship between egg size and survival probability, although the effect of egg size on the survival probability might vary depending on environment condition [41]. Therefore, by combining (Eq. 1) and (Eq. 2), the potential fitness of each morph at latitude *l *is given as

(3) f(i,l)=sici exp[di(bi+ail)].

The potential fitness for the two morphs showed different curves, which crossed at the northern latitude of ca. 36 degrees. The potential fitness of gynomorphs was higher in the south and lower in the north than that of andromorphs (Figure 2d), suggesting differential reaction norms between morphs (gene-by-environment interaction).

### Prediction of morph frequency cline

We calculate the frequency of andromorphs with and without NFDS, assuming the latitudinal cline in the estimated potential fitness derived from the above morph-specific reaction norms. Without NFDS, it is evident that the morph with a higher potential fitness will dominate the other morph, irrespective of the difference in potential fitness in two morphs. This means that even the morph with a slightly higher potential fitness will become fixed in each population, and that one morph is fixed randomly even if the potential fitnesses of the two morphs are exactly identical. Therefore, the latitudinal cline in potential fitness becomes the threshold reaction in which one morph shift to the other at the boundary of equal potential fitness in the absence of other evolutionary forces antagonistic to local selection derived from the differentiation of potential fitness. On the other hand, NFDS that is independent from the potential fitness may counteract a certain amount of difference in the morph specific potential fitness. Here the cline of the difference in potential fitness in two morphs is replaced by the cline in morph frequency as long as the difference in potential fitness is counteracted by NFDS, as follows.

Here, we have to consider NFDS, i.e., frequency-dependent male sexual harassment [33,34], in order to estimate "realized fitness" in the wild. Such harassment decreases the daily number of eggs laid and longevity (reproductive lifespan), both of which are the important components of "realized fitness". We supposed that fitness reduction due to male harassment is frequency-dependent, though fitness-related morphological traits are frequency-independent because these traits are determined by developmental performance during their larval stages. For simplicity, we assumed that the rate of fitness reduction (*r*(*p_i_*)) for each phenotype decreased linearly with the frequency (*p_i_*) of that phenotype [34], such that

(4) $$r\left(p_{i}\right)=1-hp_{i},$$

where the constant parameter *h *(0 ≤ *h *≤ 1) represents the intensity of male harassment. A high value of *h *indicates a drastic reduction in fitness. Therefore, combining (Eq. 3) and (Eq. 4), the "realized fitness" for each morph is given by

(5) $$F_{i}\left(p_{i},l\right)=f\left(i,l\right)r\left(p_{i}\right).$$

The evolutionary equilibrium frequency p_i_* was calculated by setting *F*_A _= *F*_G_, where *p*_A _+ *p*_G _= 1.

**Figure 3:**
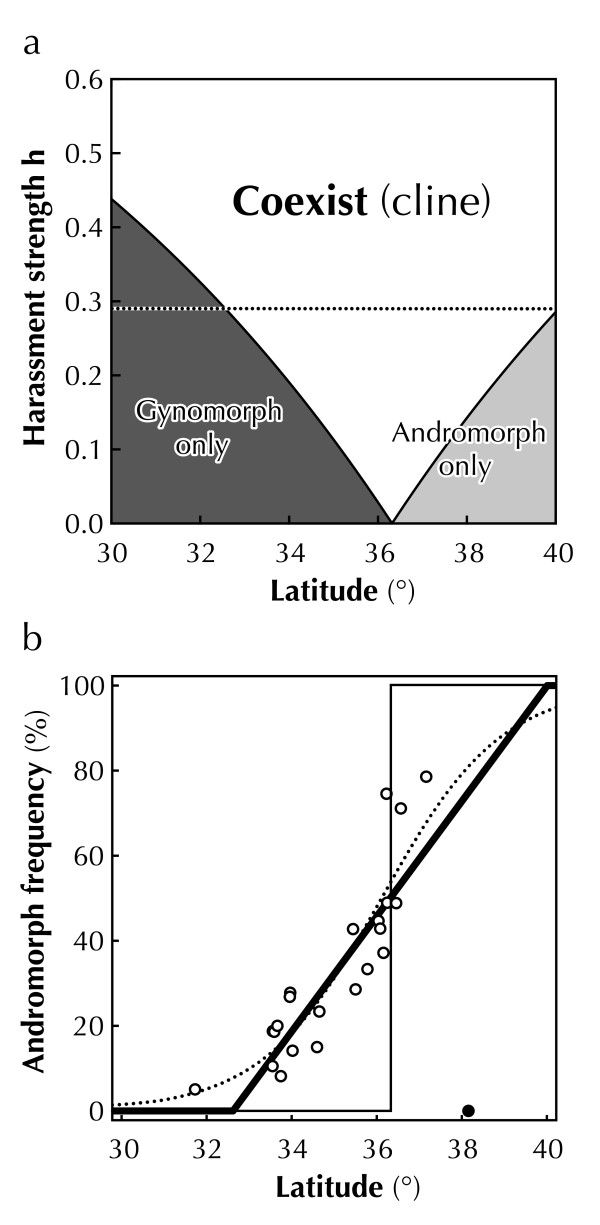
**Estimated cline in morph frequency**. (*a*) Phase diagram of morph-frequency equilibrium with latitude *l *and harassment strength *h*. Equilibrium frequency was calculated from the equilibrium conditions (*F*_A _= *F*_G_) for equation (5). (*b*) The estimated cline (thick solid line; *h *= 0.288) is shown with the observed cline (data points with dashed line from Figure 1) and a steep cline (thin solid line; *h *= 0). The level of male harassment *h *that correctly predicts the width of the morph frequency cline in the wild (*h *= 0.288, horizontal dashed line in Figure 3a) was calculated by using the least-square fitting method (*r*^2 ^= 0.77, adjusted *r*^2 ^= 0.76).

Figure 3a shows the phase diagram of equilibrium states for latitude *l *and harassment strength *h*. Using the estimated relationship between latitude and fitness potential of female morphs, equilibrium states were calculated under the various strength of NFDS and classified into three phases: andromorph only, gynomorph only and coexist. On the boundary between each phase, disadvantage in the fitness potential of certain morphs is countered by rare morph advantage when its frequency is juts zero. When the effect of NFDS was absent (*h *= 0), the andromorph frequency at the equilibrium point changed discretely from 0 to 1 at ca. 36°, where each morph exhibited the same potential fitness (*F*_A _= *F*_G_), resulting in a stepwise cline (Figure 3a). However, in the presence of NFDS (*h *> 0), a smooth cline with latitude was established. Because NFDS allows the coexistence of female morphs in each local population even when the potential fitness differed between morphs, equilibrium frequencies, where each morph has equivalent "realized fitness", were determined by the degree of difference in potential fitness between morphs. Since relative difference in potential fitness between morphs changed gradually with latitude (Figure 2d), cline morph in frequency was predicted to be smooth under constant NFDS. The equilibrium frequency was gynomorph biased in the low-latitude populations, and andromorph biased in the high-latitude populations (Figure 3a).

The slope of the cline (i.e., cline width) depends on parameter *h *(Figure 3a). That is, the smooth geographic cline was established when male harassment (i.e., NFDS) was severe. Irrespective of the harassment strength *h*, the estimated morph frequency became 0.5 (equilibrium point) at ca. 36° latitude. This estimated equilibrium point (36°) agrees well with that observed in the wild (Figures 1,3b). Using the least-square fitting method, the level of male harassment *h *that correctly explained the width of the morph frequency cline in the wild was 0.288 (*r*^2 ^= 0.77, adjusted *r*^2 ^= 0.76) (Figure 3a,b).

We also calculated the predicted latitudinal cline of the morph frequency, assuming that the fitness reduction rate (*r*(*p_i_*)) for each phenotype non-linearly increases with the frequency (*p_i_*) of own phenotype [34], such that

$$r\left(p_{i}\right)=\exp\left[-h_{n}\;p\right]$$

where the constant parameter *h *(0 ≤ *h_n _*≤ 1) represents the intensity of male harassment. The predicted latitudinal cline (Figure not shown) did not differ qualitatively for original prediction that assumes linear relationship between morph frequency and the cost of male harassment (Figure 3a,b).

## Discussion

Most coenagrionid damselflies are extremely sedentary, with dispersal limited to the area of contiguous habitat, and their lifetime movement was reported to be ca. 50 m [21]. Molecular studies of damselflies revealed that local populations tend to be isolated because their habitats (ponds) are located far relatively far apart [22,42]. In the current system, geographic gene flow (dispersal) cannot be expected, as indicated by previous studies [22] and the current isolated population in the northern end (gynomorph only). Therefore, it is unlikely that gene flow alone causes the smooth geographic clines observed over several hundred kilometers in some polymorphic damselflies [18-20]. In the current study, we identified a large-scale (over 1100 km) smooth cline in female morph frequency in *I. senegalensis*. We also showed that the current latitudinal cline in morph frequency is well explained by the combination of NFDS [34] and the differing potential fitness between morphs (gene-by-environment interaction) without the effect of gene flow. Note that current prediction of the location of the cline using independent field data (Figure 2a-d) agrees with the observed latitudinal cline in the wild (Figure 3a), and NFDS can explain the geographic cline in morph frequency in this system (Figure 3a,b). The current study indicates a multi-selection pressure underlying geographic clines in morph (or allele) frequency in real examples in the wild.

In some polymorphic species, morph frequency cline does not extend beyond an equilibrium point (1:1) due to limitation of their distribution range [15]. Gene flow cannot explain the establishment of such cline, because genes that are adaptive at the opposite side of equilibrium are never supplied. However, balancing selections can explain establishing such clines, because the combination of gene-by-environment interaction and balancing selection independently determine the equilibrium morph frequency in each local population. Even when potential fitness in a given population differs between morphs, polymorphism is protected in the population if the advantage of fitness potential of certain morphs is countered by frequency-dependent disadvantage (common morph disadvantage). The larger the difference in fitness potential between morphs becomes, the more the equilibrium of morph frequency biases. Because the difference in fitness potential between morphs gradually changes with environmental gradient, the equilibrium of morph frequency therefore changes gradually, resulting in a smooth morph frequency cline. Balancing selections are more important for establishing morph frequency cline than we might have expected.

Gene-by-environment interaction effect of fitness is commonly observed in polymorphic organisms. For example, melanic morphs in the butterfly *Colias eurytheme *entail adaptive advantage in the cold, but non-melanic morphs are more adaptive than melanic morphs in warm condition as it has a less tendency to overheat [43]. The relative fitness of two color morphs in *Colias *butterfly thus depends on the environmental conditions. On the other hand, morph-specific developmental reactions to environmental condition may also result in gene-by-environment interactions. In female-polymorphic damselflies, female morphs differ in a variety of traits, such as larval development [44] and body size [45], due to correlational selection, which favors adaptive trait combinations [46,47]. As a result of the integration of multiple traits, color morphs may show differing reaction norms to environmental conditions [48-50]. In *I. senegalensis*, the potential fitness of the two female morphs reversed with latitude.

In the plant species *Narcissus triandrus*, NFDS was also attributed to the geographic variation in flower morph frequency [8]. In this case, reproductive interaction between flower morphs that governs NFDS changed geographically. Therefore, the changes in NFDS itself created geographical variation in morph frequency. In contrast, in the current damselfly system, the combination of consistent NFDS and the morph-specific clinal variation of potential fitness can explain the geographic cline in morph frequency along latitude without gene flow.

There is another important aspect in current study for the maintenance of geographic cline in morph frequency. We found an underlying natural selection that is necessary to cause a cline in morph frequency (i.e., difference in potential fitness between the morphs) and imbedded this factor into the model for maintenance of genetic dimorphism. In the current system, NFDS alone is not sufficient to form the cline in morph frequency and the geographic morph cline cannot be expected without the underlying potential fitness difference between the morphs. Thus, multiple selection pressures acting on one trait may dampen the speed at which a morph with higher fitness becomes fixed or balance the fitness at different optima. Indeed, the combination of NFDS and introgression [20] and the combination of NFDS and morph specific thermal tolerance [12,16] is suggested to establish morph frequency cline. In other case, environmental heterogeneity and gene flow are likely to jointly influence the large-scale geographic patterns in morph frequencies. Multiple microevolutionary forces are necessary to cause a smooth clinal variation in polymorphic system [18].

The pattern of clinal variation in body size (i.e., fecundity) is consistent with Bergmann's rule, in which body size tends to be bigger in colder climates (e.g., [51-54]). It is explained by either (A) a physiological constraint relating to temperature or (B) adaptation to ambient temperature in ectothermic animals [55].

In the former (A), the individual body size of ectotherms tends to decrease with increasing temperature due to a physiological constraint (temperature-size rule) [56]. The growth rate controlled by protein systhesis is less sensitive to temperature, but the rate of cell division is highly sensitive to temperature [57]. Therefore, body size becomes smaller with higher temperatures. For example, Daufresne et al. [54] reported that the body size of aquatic ectotherms decreases in response to climate warming. Since the larvae of damselflies are aquatic, their body sizes may also becomes smaller in the south (see Figure 2).

In the latter (B), the fitness optimum is assumed to lie at a small body size at a warm temperature. Under direct solar radiations, a large body increases its body temperature more effectively [58]. This results in a large body in the north and a small body in the south. The small body may also effectively avoid over-heats in the south. If the latter explanation is true in the current damselfly, the potential fitness might have to be re-examined with the consideration for the size-dependent thermoregulation. Common garden experiments may show which explanation is appropriate by revealing the relationship between ambient temperatures and larval developments.

## Conclusion

The geographic scale cline in the morph frequency in *I. senegalensis *is suggested to be the result of a balance between two conflicting selection pressures: local selection and NFDS. Differing reaction norms to the environment between morphs [49] as well as balancing selection (e.g., NFDS) may underlie many polymorphic systems in both animals and plants [46,59,60]. Therefore, balancing selection may strongly contribute to establishing a geographic-scale cline in morph (allele) frequency along environmental gradient in nature. In the current system, the two morphs have different potential fitness along the latitudinal gradient. The andromorph (gynomorph) has a higher potential fitness in the north (south) and the potential fitness becomes equivalent at around 36°. Without NFDS, the andromorph (gynomorph) wins completely in the north (south) and the two morphs will switch at the 36°. However, with NFDS, the low potential fitness of a rare morph is compensated to some extent by the advantage of rarity. For example, at the 34°, the andromorph (ca. 0.2) has the advantage of rarity, while the gynomorph (ca. 0.8) has the higher potential fitness. The actual fitness (overall fitness) of the two morphs becomes equivalent, resulting in the stable coexistence. Thus in the wide range of latitudinal gradient, the coexistence of the two morphs becomes possible (Figures 1,3).

We, in the current study, ruled out the effect of gene flow, and showed that NFDS was sufficient to explain the geographic-scale cline in morph frequency. However, it is common knowledge that there is gene flow in a flying insect [23-25]. Although Endler [6] demonstrated that morph frequency cline along environmental gradients is independent of the effect of gene flow when balancing selection is acting, relatively high dispersal compared to local selection may affect geographic cline in the morph frequency. Even short-distance dispersal may contribute to provide continuity to morph frequency cline by floating the micro-variation of morph frequency among neighboring populations. Therefore, to confirm the role of NFDS in maintaining a smooth cline, some additional works quantifying the dispersal rate and its contribution by using mark-recapture method or molecular analyses are needed. The relative importance of NFDS and gene flow in establishment of geographic cline in morph frequency should be tested for each polymorphic system.

The maintenance of dimorphic clines is still not understood well in almost all cases. Even though it may not be the only explanation, our study shows that NFDS is at least sufficient to explain the geographic cline in morph frequency with no gene flow (Figure 3a,b). The current study provides the suggestive evidence for the contribution of NFDS to geographic clines in morph (or allele) frequency in real examples in the wild. Our simple model roughly but correctly predicted the pattern of the geographic cline of morph frequency, although we made some assumptions and ignored some fitness components, such as survival rate and developmental time. These fitness components might depend on environmental conditions, which varied among populations. The validity of these assumptions and the contribution of other fitness components should be tested to explain the detailed inter-population variation in the morph frequency at small instead of at geographic scales.

## Methods

### Study species and study sites

*Ischnura senegalensis *is a non-territorial damselfly that inhabits the open and sunny edges of ponds. Whereas males are monomorphic, females exhibit color dimorphism: an andromorph and a gynomorph [34,61]. The female color morphs are determined by two alleles at a single autosomal locus with sex-limited expression. The allele for andromorph is recessive to that of gynomorph, similarly to other female-dimorphic damselflies [62,63].

We conducted field surveys of 22 local populations (north latitude: 31-38°) in Japan between April and June 2009 (spring generation) (Additional file 1 Table S1). To ensure data independence, we only sampled populations that were more than 10 km apart. The northernmost population was sampled from a region that is presumably the northern limit of distribution range in *I. senegalensis*. For all the local populations, latitude and longitude were determined using of Google Earth.

### Morph-frequency variation in the wild

Morph frequency was recorded using the line transect method in 22 local populations. Census lines (200-500 m) were set along the edge of the ponds. Each line census was carried out in the morning by walking slowly along the line so as to avoid disturbance. To estimate morph frequency, we recorded the number of males and the two female morphs found within 1 m on both sides of the line. The mean number of females detected ± SE in each line census was 80.7 ± 13.2. When a population was sampled more than once (at different times in the spring generation), the mean of the replicates was used as the morph frequency.

### Latitudinal variation in potential fitness

In damselflies, because no differences in survival rate has been found between morphs (e.g., [64]), the ability of egg production is the best indicators of "potential fitness (potential fitness advantage)" [28,36], and is generally restricted by the number of ovarioles [35,37,65]. Thus, we assume that the number of ovarioles represents the total number of eggs produced for a given morph. However, we could not directly assess the number of ovarioles in the females sampled because the ovarioles broke apart when fixed with ethanol. For each morph, the number of ovarioles correlates with the length of the abdomen, and the length of abdomen correlates with latitude (see below). Therefore, we estimated the number of ovarioles for each morph at a given latitude from these two relationships. Because egg size also differed between morphs, the potential fitness was calculated from the number of ovarioles and egg size. In the current study, we estimated the potential fitness of females in each local population using wild individuals, assuming that the morphological traits relating to female potential fitness such as body size (the number of ovarioles) are independent of male sexual harassment. This assumption should be valid since these morphological traits are determined by developmental performance during their larval stages.

To examine morph-specific latitudinal changes in the length of the abdomen, adults were sampled from 12 local populations (see Additional file 1 Table S1). The adult samplings were carried out in the early breeding season of each population because body size might change with time during the breeding season. A slide caliper was used to measure the length of the abdomen in the females captured. The females were dissected under a stereomicroscope, and the length (*L*) and width (*W*) of mature eggs were measured with a micrometer using three eggs randomly sampled from each female. The means of each trait in each population were used to eliminate any effects of pseudoreplication.

To investigate the relationship between the length of the abdomen and the number of ovarioles, a sampling of each female morph in population O was carried out on 25 May 2009 (see Additional file 1 Table S1). All females were dissected under a stereomicroscope, and the total number of ovarioles in the right and left ovaries were counted (see [35]).

### Statistical analyses

Statistical analyses were performed using R version 2.9.0 (R Development Core Team, 2009). All values are presented as means ± standard error. Geographical variation in morph frequency in the wild was analyzed by logistic regression. The mean values of each trait (the length of abdomen and egg size) for each population were used to eliminate the potential effects of pseudoreplication. Geographical variations in the length of abdomen and egg size were analyzed by multiple linear regression analysis. Differences in egg size between morphs were compared using a two-sample *t*-test. The relationship between the length of the abdomen and the number of ovarioles was analyzed using a generalized linear model (GLM).

## Authors' contributions

YT and MW designed and analyzed the field experiments. YT, JY and SM constructed the mathematical models. All four authors participated in discussion of the results and contributed to manuscript preparation. All authors read and approved the final manuscript.

## Supplementary Material

Additional file 1 **Table S1**: Results of the line census in 2009. Click here for file

Additional file 2 **Figure S1**: Relationship between latitude and the volume of mature eggs for each morph. Click here for file

Additional file 3 **Figure S2**: Relationship between the length of abdomen and the number of immature eggs in an ovariole. Click here for file
