# Supplementary material for: A geographic cline induced by negative frequency-dependent selection
Source: BMC Evol Biol. 2011 Sep 14;11:256. doi: 10.1186/1471-2148-11-256 (PMC3185284; doi:10.1186/1471-2148-11-256)
Supplement: Additional file 1 — Table S1: Results of the line census in 2009. [file 1471-2148-11-256-S1.DOC]

**Additional file 1**

| **Table S1**. Results of the line census in 2009. | | | | | |
| --- | --- | --- | --- | --- | --- |
| Population | Location | Date | No. census | Andromorph frequency | SD |
| A* | 31°43'39"N, 130°39'24"E | 10, 11 May | 2 | 0.05 | 0.010 |
| B | 33°32'58"N, 130°21'47"E | 12 May | 1 | 0.11 | – |
| C | 33°33'23"N, 130°26'57"E | 13 May | 1 | 0.19 | – |
| D* | 33°35'22"N, 130°22'48"E | 13 May | 1 | 0.19 | – |
| E | 33°39'51"N, 130°25'13"E | 13 May | 1 | 0.20 | – |
| F | 33°44'43"N, 130°28'44"E | 12 May | 1 | 0.08 | – |
| G | 33°57'23"N, 131°12'37"E | 15 May | 1 | 0.27 | – |
| H | 33°57'56"N, 131°13'24"E | 14 May | 1 | 0.28 | – |
| I* | 34° 1'18"N, 131°22'49"E | 15 May | 1 | 0.14 | – |
| J* | 34°35'44"N, 133°55'35"E | 30 May | 1 | 0.15 | – |
| K* | 34°39'1"N, 133°57'33"E | 28, 29 May | 2 | 0.23 | – |
| L* | 35°26'34"N, 133°17'12"E | 31 May; 1 June | 2 | 0.43 | 0.055 |
| M | 35°30'7"N, 133°14'33"E | 31 May | 1 | 0.29 | – |
| N* | 35°46'53"N, 139°52'57"E | 7 June | 1 | 0.33 | – |
| O* | 36° 2'29"N, 140° 8'51"E | 7, 18, 23 May; 4, 23 June | 5 | 0.45 | 0.046 |
| P | 36° 4'52"N, 140° 4'51"E | 18 May | 1 | 0.43 | – |
| Q | 36° 9'28"N, 140° 3'48"E | 7, 18, 20 May; 6 June | 4 | 0.37 | 0.052 |
| R* | 36°13'43"N, 136°10'52"E | 9 June | 1 | 0.75 | – |
| S | 36°14'40"N, 140°19'10"E | 20 May; 3 June | 2 | 0.49 | 0.079 |
| T* | 36°27'25"N, 140°35'42"E | 20 May; 3 June | 2 | 0.49 | 0.042 |
| U | 36°34'1"N, 136°39'33"E | 10 June | 1 | 0.71 | – |
| V* | 37° 9'48"N, 140°55'40"E | 12 June | 1 | 0.79 | – |
| W* | 38° 9'23"N, 140°55'40"E | 23 June | 1 | 0.00 | – |

*: populations where adult samplings were carried out
