# Supplementary material for: A geographic cline induced by negative frequency-dependent selection
Source: BMC Evol Biol. 2011 Sep 14;11:256. doi: 10.1186/1471-2148-11-256 (PMC3185284; doi:10.1186/1471-2148-11-256)
Supplement: Additional file 2 — Figure S1: Relationship between latitude and the volume of mature eggs for each morph. [file 1471-2148-11-256-S2.DOC]

**Additional file 2**


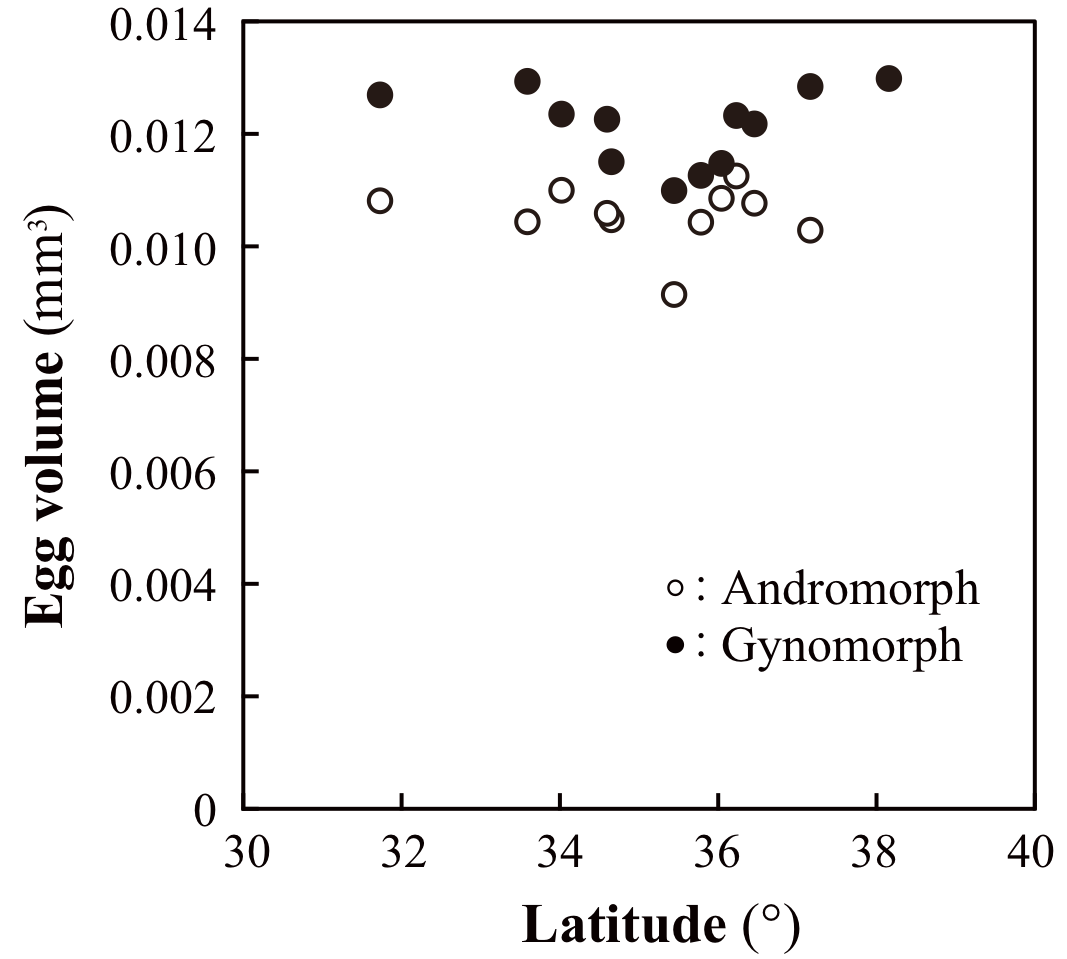


**Figure S1**. Relationship between latitude and the volume of mature eggs for each morph. Each data point represents the morph average of a population. We find no significant correlation between the latitude and egg size (*t* = –0.277, *df* = 22, *P* = 0.785), but the effect of morph on egg size was significant (*t* = 6.024, *df* = 22, *P* < 0.001).
