# Supplementary material for: A geographic cline induced by negative frequency-dependent selection
Source: BMC Evol Biol. 2011 Sep 14;11:256. doi: 10.1186/1471-2148-11-256 (PMC3185284; doi:10.1186/1471-2148-11-256)
Supplement: Additional file 3 — Figure S2: Relationship between the length of abdomen and the number of immature eggs in an ovariole. [file 1471-2148-11-256-S3.DOC]

**Additional file 3**


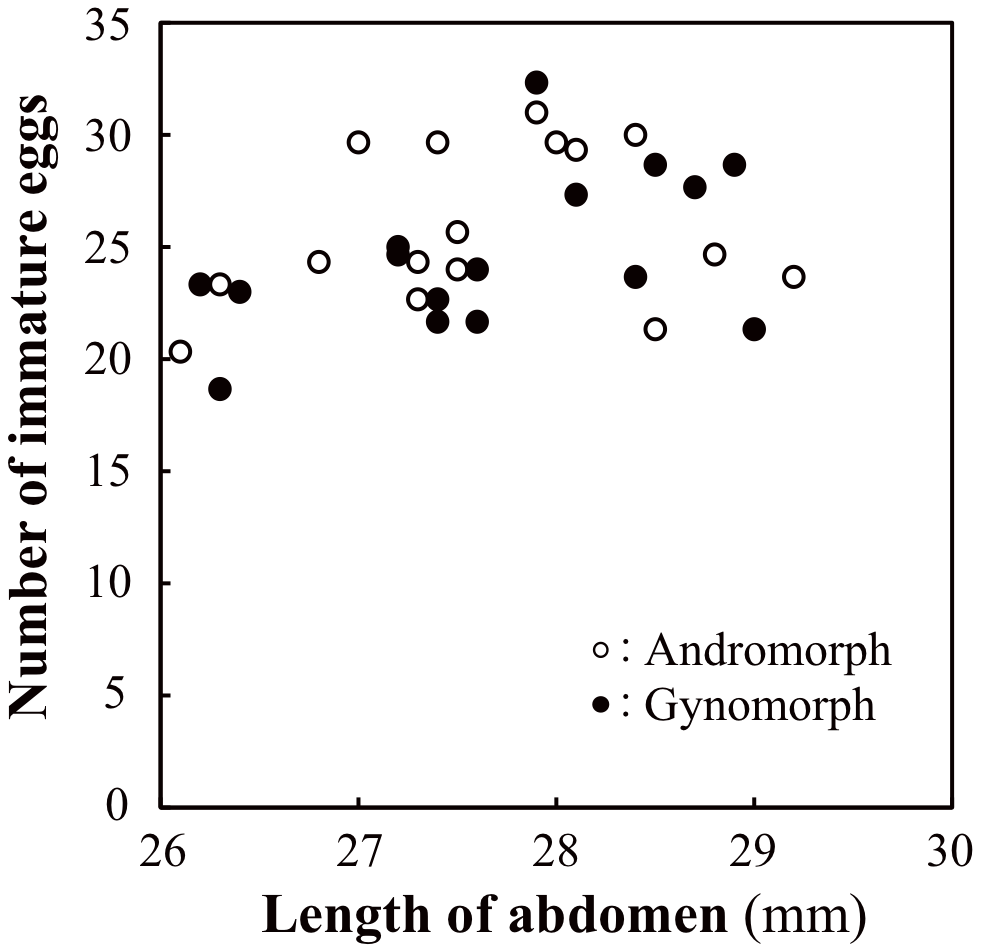


**Figure S2**. Relationship between the length of abdomen and the number of immature eggs in an ovariole. For sexually immature females sampled on 25 May 2009 in “population O,” the number of immature eggs in each ovariole was counted in based on three ovarioles randomly sampled (see Ref. S1). There are no significant differences in the number of immature eggs in an ovariole between andromorphs (25.9 ± 0.89, *N* = 16) and gynomorphs (24.6 ± 0.87, *N* = 16) (two-sample *t*-test, *t* = 0.943, *df* = 30, *P* = 0.35). There is no relationship between the length of abdomen and the number of immature eggs in an ovariole (*t* = –0.721, *df* = 31, *P* = 0.477). The effect of morph and the interaction effect of between the length of abdomen and morph are also insignificant (*t* = –0.721, *df* = 31, *P* = 0.477, *t* = 0.688, *df* = 31, *P* = 0.497, respectively).

**ADITTIONAL REFERENCE**

S1. Takahashi Y, Watanabe M: **Morph-specific fecundity and egg size in the female-dimorphic damselfly *Ischnura senegalensis*.** *Zool Sci* 2010, **27:**325–329
